# X-ray Spectroscopy Fingerprints of Pristine and Functionalized Graphene

**DOI:** 10.1021/acs.jpcc.1c03238

**Published:** 2021-08-16

**Authors:** Anja Aarva, Sami Sainio, Volker L. Deringer, Miguel A. Caro, Tomi Laurila

**Affiliations:** † Department of Electrical Engineering and Automation, School of Electrical Engineering, Aalto University, 02150 Espoo, Finland; ‡ Stanford Synchrotron Radiation Lightsource, SLAC National Accelerator Laboratory, Menlo Park, California 94025, United States; § Microelectronics Research Unit, Faculty of Information Technology and Electrical Engineering, University of Oulu, P.O. Box. 4500, 90570 Oulu, Finland; ∥ Department of Chemistry, Inorganic Chemistry Laboratory, University of Oxford, Oxford OX1 3QR, U.K.; ⊥ Department of Chemistry and Materials Science, Aalto University, Kemistintie 1, 02150 Espoo, Finland

## Abstract

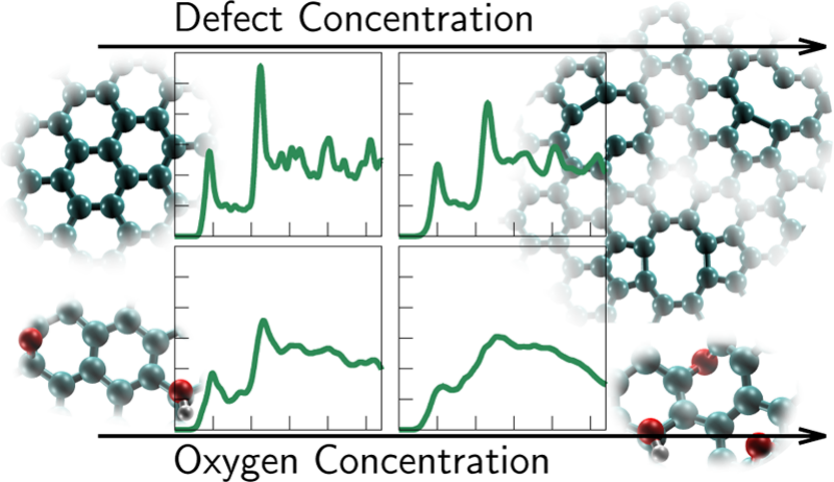

In this work, we demonstrate how to identify and characterize the atomic structure of pristine and functionalized graphene materials from a combination of computational simulation of X-ray spectra, on the one hand, and computer-aided interpretation of experimental spectra, on the other. Despite the enormous scientific and industrial interest, the precise structure of these 2D materials remains under debate. As we show in this study, a wide range of model structures from pristine to heavily oxidized graphene can be studied and understood with the same approach. We move systematically from pristine to highly oxidized and defective computational models, and we compare the simulation results with experimental data. Comparison with experiments is valuable also the other way around; this method allows us to verify that the simulated models are close to the real samples, which in turn makes simulated structures amenable to several computational experiments. Our results provide *ab initio* semiquantitative information and a new platform for extended insight into the structure and chemical composition of graphene-based materials.

## I Introduction

Graphene (G) and graphene oxide (GO) have attracted the attention of academic research as well as industry globally, in particular since 2010.^1,2^ Graphene-based materials are promising candidates for a vast variety of applications in several fields, such as biotechnology, nanoelectronics, solar cells, lithium-ion and sodium-ion batteries, supercapacitors, anticorrosion coating, and sensors, to name a few.^3^ This growing interest led, for instance, to the European Union launching in 2013 the Graphene Flagship research program, funded with 1 billion EUR.^4^

Many of the current scientific endeavors focusing on graphene and derivatives promise to bring this material and its outstanding properties (mechanical, thermal and electrical/electronic) from the laboratory to industry. These efforts are hindered by the lack of detailed understanding of the atomic structure of graphene-based materials, beyond the most simple ones, such as pure sp^2^-bonded crystalline graphene and graphite. Carbonaceous materials often also contain elements other than carbon, especially oxygen functionalizations, in many forms. Ideally, graphene would consist of monolayered sp^2^-bonded carbon only, but in the experimental reality this is often not the case. When we move from the 2D graphene structure (including defects, doping and impurities, whether intentional or not) to graphite, the structure is still sp^2^-rich, but the complexity of the material is again increased. Previously, experimental X-ray spectroscopy has been utilized in order to understand the structure of GO,^5,6^ but since the number of experimental samples has been quite limited and, in addition, sample preparation methods as well as the precursor materials differ, it is hard to compare the results. So what do we really know, in practice, about the structure–property relationships of these materials?

The actual structure of GO, not to mention graphite oxide, has been under debate for some time now. In 2010, Dreyer et al. published a Critical Review^7^ about synthesis and structure of GO. Their main conclusion is that there is no single GO but that the structure, properties, and nature of GO depend strongly on the quality of the precursor material, i.e., the graphene or graphite source, as well as the oxidation protocol.^7^ This conclusion is readily justified, since the complex chemistry involved can yield several kinds of outcomes. The same logic applies for many carbon allotropes. Also, more recent voices have been raised for the importance of understanding the relationship between the performance and experimental characterization of this material.^8,9^ In this work, we include both GO and reduced GO (rGO), in the form of samples that contain different amounts of oxygen-containing functional groups. In other words, we study a range from ordered, pristine, or precisely functionalized materials to nearly amorphous structures. Careful characterization is the key for understanding the link between the structure and properties of GO.

In this study, we provide a computational methodology that extends on initial work in refs (10−13), as well as a comprehensive set of reference data, for interpreting experimental X-ray spectroscopy data of graphene-based compounds, aiming at careful structural and chemical characterization of these materials. Among the different X-ray spectroscopy techniques, we focus on X-ray absorption spectroscopy (XAS) and X-ray photoelectron spectroscopy (XPS). XAS and XPS are popular and accurate methods for analyzing the composition of materials in general.^14,15^ XAS probes the allowed transitions from electronic core levels to conduction (unoccupied) states. In other words, it provides detailed information about the structure of the material’s conduction band. XPS is a more widely used method. It provides the spectrum of core–electron binding energies. However, especially in the case of structurally and chemically complex materials, as is often the case for GO, interpreting the experimental data is extremely challenging due to the features arising from varying chemical environments. Recreating the spectra from first-principles can be an invaluable aid toward understanding the highly convoluted experimental data. Several steps, on different levels of theory, for computational interpretation of XAS and XPS spectra have already been taken,^10,11,16−29^ and now it is time to turn the focus onto systematic analysis of graphene-based materials.

We use a carefully selected ensemble of model structures, to represent the different existing types of graphene-based materials. From these structures, we calculate their signature X-ray spectral responses from density functional theory (DFT). These *fingerprint spectra*(10) are then reclassified according to the immediate chemical environment of the atomic sites from which they originate, using unsupervised machine learning (ML). These calculated spectra can then be compared with experimental spectra via computational fitting,^11^ to estimate the type and composition of the experimental graphene/graphite sample in question. In this way, we manage to provide a qualitative and quantitative means to characterize the range of atomic structures present in G- and GO-based materials.

## II Computational and Experimental Protocols

**Figure 1:**
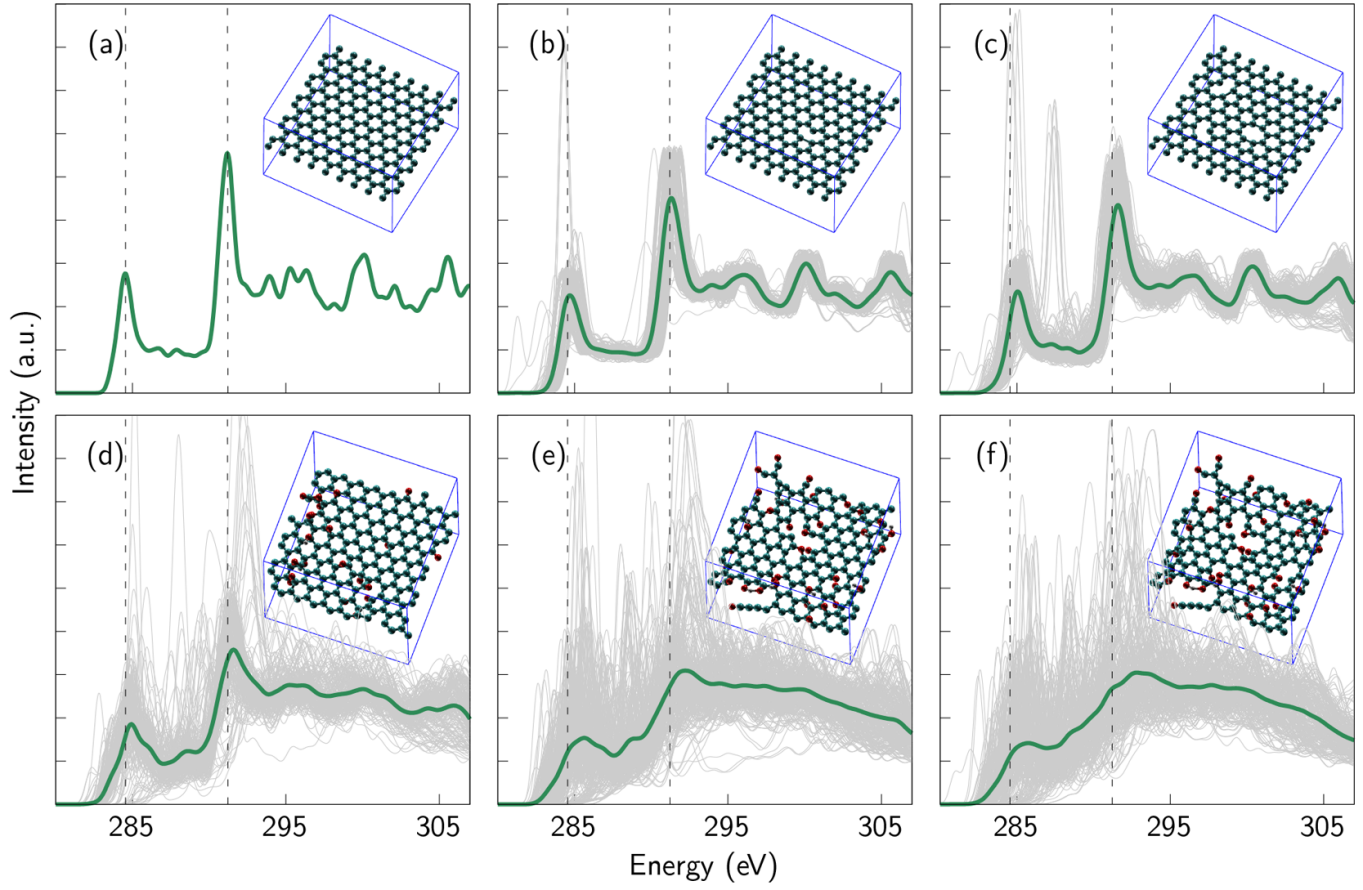
Simulated C 1s XAS spectra of graphene samples when defect and/or oxygen concentration is gradually increased: (a) pristine graphene, (b) graphene with a single vacancy defect, (c) defective sample with high vacancy concentration, (d) graphene with some oxygen, (e) graphene with more oxygen, and (f) graphene with high oxygen and defect concentration. The upper row consists of samples without oxygen, whereas the oxygen-containing samples are placed in the lower one. The oxygen concentration varies from 10 at. % up to 19 at. %. After the pristine sample, the defect concentration varies systematically from one SV defect to four missing carbon atoms, although some samples showed tendency for self-healing, i.e., vacancies were closed during relaxation, which lead to the presence of some disordered ring structures (Section III). The corresponding spectra change from representing pristine graphene to something that is nearly amorphous. The main pristine graphene peak positions are depicted with dashed lines as references. Calculated individual spectra of the sites in the samples are depicted with gray lines. It is clear how disorder increases as the number of inequivalent local chemical environments is varied. Schematic images of the corresponding structures are presented next to the spectra. Spectrum was reproduced with permission from ref (11). Copyright 2019 American Chemical Society. Spectra very similar to spectra b and d have also been published in ref (30), which discusses trends of carbon-based materials in XAS measurements from the experimental point of view.

In this study, we investigate the role of the defect concentration of several computational models, in the form of vacancies and oxygen-containing groups, to study trends in their X-ray spectroscopic signatures. The changes that take place when the structural models are modified, going from pristine to nearly amorphous, are depicted in Figure 1. Clearly, the dominant effect is an increasing “smearing” of the well-defined spectroscopic features of the crystalline sample as more defects are introduced. Before going into the detailed analysis of the connection between spectra and structures in Section III, this section introduces the methodologies used for obtaining the structural models, computing the spectra, doing the data classification and carrying out the experimental measurements.

### II.A Carbon-Based Structural Models

Pristine graphene, single vacancy (SV), double vacancy (DV) and multiply defective graphene structural models (Figure 1a–c and Figure 3) were made in house, using established methods, i.e., via lattice parameter optimization and relaxation. Similar structures (pristine graphene and SV) have been employed in our previous work,^10,11,30,34^ but the structural optimization and several tests were repeated for this study with a newer version of the GPAW code,^35^ to ensure methodological consistency with the new calculations.

**Figure 2:**
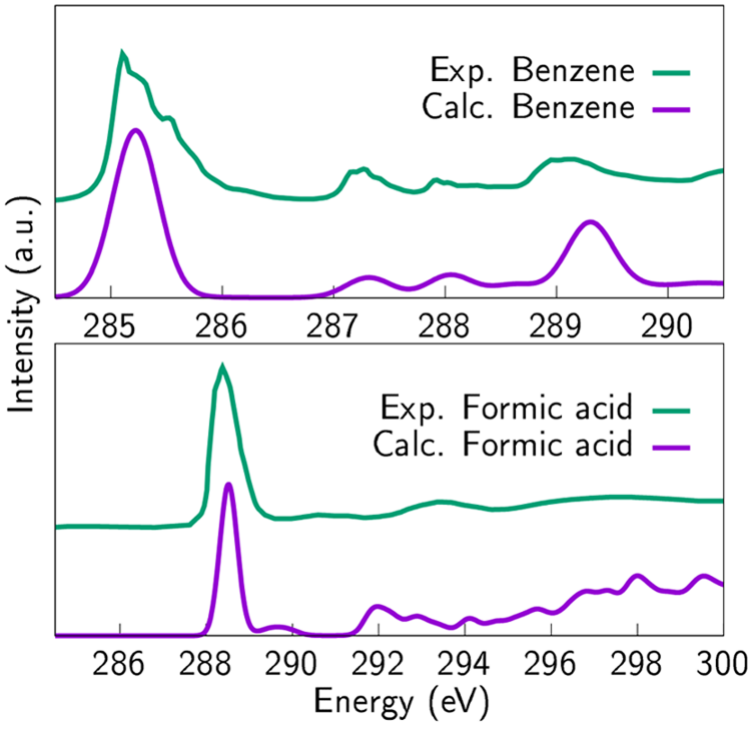
Experimental spectra of well-known molecules: benzene and formic acid, compared with computational spectra. Experimental spectra are reproduced with permission from refs (31,32), respectively. Copyright 2004 Elsevier and 2001 American Chemical Society, respectively. Note that the experimental formic acid spectrum is measured on copper substrate, which alters the result compared to the computational mode however, the agreement between the spectra is notable.

**Figure 3:**
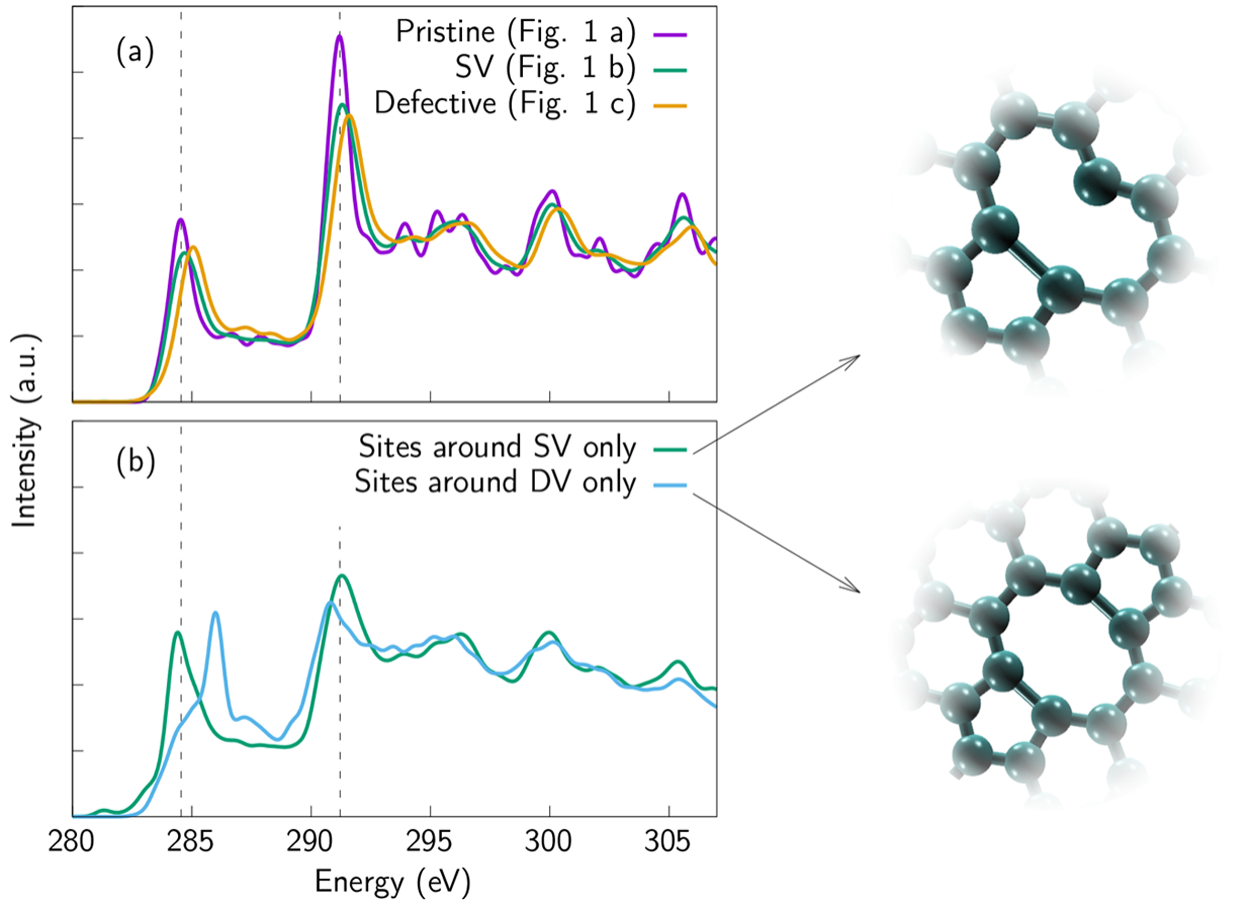
(a) Simulated XAS spectra of defective graphene samples without functionalization. The depicted spectra are averages calculated from the whole sample, except in the case of pristine graphene, since in that sample all the sites are symmetry equivalent. Corresponding structures are depicted in Figure 1. As in the case of Figure 1, the pristine spectrum has been published before in ref (11) and the SV spectrum in ref (30). Reprinted with permission. Copyright 2019 and 2020 American Chemical Society. Note that the calculated spectra are depicted as they appear, and they are not shifted according to any literature reference. (b) Fingerprint spectra of the defects: SV and DV. Only the sites around the defect were taken into account when the average spectra were calculated. Schematic images of SV and DV are depicted next to the plot.

**Figure 4:**
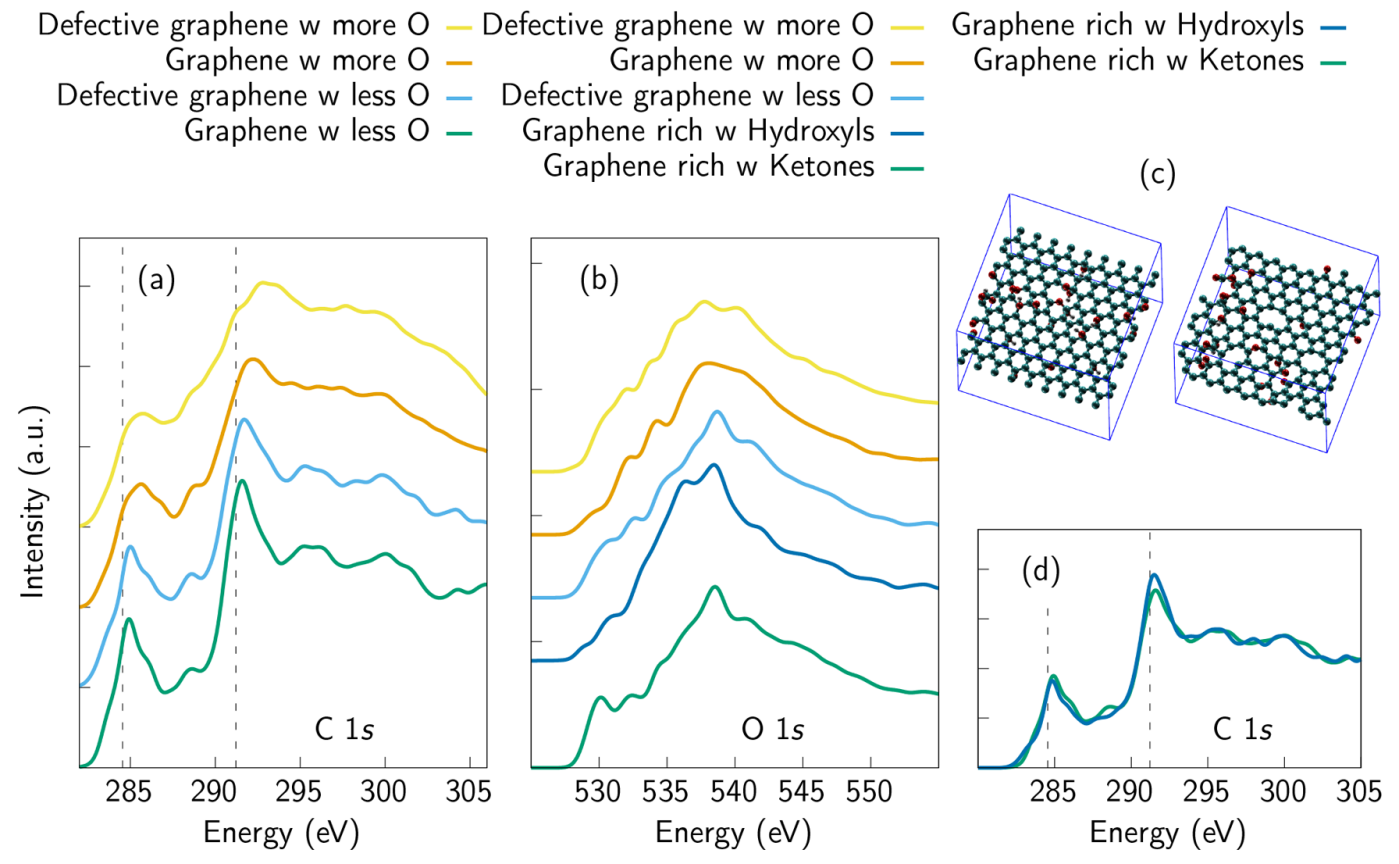
Simulated (a) C 1s and (b) O 1s XAS spectra of graphene samples with different oxygen concentrations ranging from 10 at. % up to 19 at. %. The depicted spectra are averages calculated from the whole sample and the corresponding structures are depicted in Figure 1 as well as here (c) ketone-rich (left) and hydroxyl-rich (right) next to the spectra (d) to highlight the difference between the samples.

The oxygen-containing structural models have been taken from ref (36) (Figure 1d–f and Figure 4). The oxygen concentrations shifts from 10 at. % up to 19 at. %. We performed additional cell-shape optimization and relaxation on those samples, in order to ensure consistency with the generation method and the level of theory used for our pure carbon samples. Although we have previously used a ML-based interatomic potential^37^ for efficient structure generation of amorphous carbon samples,^10,11,38−40^ this potential is limited to elemental carbon. Therefore, we rely on DFT-based functionalization for the time being following refs (38 and 39), but we envision that we will be able to expand the compositional and configurational space spanned by the present work in the near future, as reliable carbon–hydrogen–oxygen (CHO) ML potentials become available.

The monolayered systems used here consist of 176–213 atoms and periodic boundary conditions (PBC) were applied during relaxation. The system size and the convergence of excited state calculations were carefully studied before carrying out the calculations on larger scale. All calculations in this work were carried out with the DFT code GPAW,^35,41^ using the PBE functional,^42^ and van der Waals corrections as introduced by Tkatchenko and Scheffler.^43^

### II.B X-ray Spectra Calculations

To explain experimental results, we employ DFT-based simulations of XAS and XPS spectra, all performed on the structural models described above. XAS calculations are carried out as implemented in the GPAW code^35^ by Ljungberg et al.^44^ While different approximations to computational X-ray spectroscopy may not always yield satisfactory results,^17^ the GPAW implementation has been shown to perform particularly well for systems containing carbon and oxygen, and it has been shown to produce XAS spectra that are in good agreement with experiment.^10,11,44,45^ Additionally, the method has been validated by testing it with smaller molecules; benzene and formic acid (Figure 2). The first one has been a candidate for testing also previously,^20^ and the latter one shows how the sharp peak position is also related to carboxylic acid that is anticipated to be present in these substances. The experimental spectra are from refs (31 and 32), respectively. General information about excited state calculations is available in refs (20 and 46).

XAS calculations within this framework consist of two steps. First, the cross section for the transitions between the core level and the different conduction band states is obtained via the Haydock recursion method. Second, a so-called Δ Kohn–Sham (ΔKS) calculation is carried out to estimate the energy differences between the ground state and the lowest excited state (i.e., the excited state for which the core electron is promoted to the system’s Fermi level). This provides an accurate estimate of the correct energy alignment of the XAS spectra. In addition, there is a correspondence between the ΔKS values and experimental XPS spectra. Further details about the method are given in refs (44 and 47), and its applicability to carbon-based systems has been covered in detail in the literature.^23,48,49^ We particularly refer the reader to our previous work on amorphous carbon.^10,11^

Compared to our previous work,^10,11^ we have introduced some improvements in the methodology, especially for more consistent handling of the magnetic structure (local magnetization) that arises in the presence of defects and/or amorphous structures.^8,51^ Even for crystalline materials, local magnetization effects appear in the presence of a core hole.^44^ For the present study, we carry out five self-consistent-field (SCF) DFT calculations for the ground state energy of each structure. Each of these calculations starts with a different (random) initialization of the local magnetic moments (local spins). For systems with complex magnetic structure, such as highly disordered carbons (the case here), each of these calculations may converge to a different metastable ground state. Typical differences in these energies range from negligible (μeV) up to 100 meV or so. The lowest among these five energies is selected as ground state energy. For the subsequent excited state calculation, the system’s total magnetic moment is fixed to the magnetic moment of the ground state plus and minus one spin, corresponding to the two possible core-state to conduction-band transitions (one where the core electron’s spin has the same sign as the valence band’s total spin, and one where it has opposite sign). In total, for each atomic site we carry out (i) five “ground state” calculations, (ii) two ΔKS calculations (“spin up” and “spin down”), and (iii) two Haydock recursion calculations for the XAS cross sections (also spin up and spin down). We note that, at nine DFT calculations per site, for a typical periodic supercell of ∼200 atoms, these calculations are about 2000 times more expensive than a regular DFT calculation. The numbers reported here are the transition energies averaged between both spins, noting that this is reasonable for condensed matter systems where the splitting is small. On the other hand, splittings can be rather large for molecular systems, such as O_2_.^52^ Nevertheless, averaging between the spin channels allows us to make sure that we are examining all possible cases that can be present in the samples.

In all cases, *k*-space integration was performed on a 2 × 2 × 1 Monkhorst–Pack (MP) grid.^53^ The simulation box dimensions along the *x* and *y* directions were approximately 22 and 21 Å, respectively. The unit cell size along the *z* direction was 15 Å when oxygen is not present in the samples. In the case of oxygen-containing samples the amount of vacuum was increased to 20 Å in order to ensure convergence, due to the presence of functional groups on both sides of the films and because the most defective structures are buckled. Because of the importance of spin effects highlighted above, all calculations were carried out with spin polarization.

In addition to the existence of local magnetic moments in disordered carbon structures, they exhibit excitonic effects due to the Coulomb interaction between the core hole and the excited core electron. There are two sides to core excitons in disordered carbons, both of which pose challenges within the context of the present methodology.^10,22^ On the one hand, the excitonic resonance in the XAS, whereby the cross section for core electron excitations is increased for transition energies corresponding to the bound exciton state (which shows up as a characteristic sharp feature in the XAS of crystalline carbon materials), cannot be reproduced.^44^ On the other hand, for the ΔKS calculations, a bound hole–electron pair forms when the core electron is removed from the core and added to the conduction band. The corresponding exciton binding energy would need to be subtracted to obtain the actual energy difference between the core state and the Fermi level (i.e., the actual core electron’s binding energy). There is currently no established procedure to carry out this correction. Fortunately, this artifact leads to a *systematic* underestimation of ΔKS energies, which is easily accounted for with a constant shift of the energy scale, as discussed in more detail in refs (10 and 22). Therefore, when experimental spectra are fitted with computational data sets, a small constant shift of all ΔKS values, i.e., the energy alignment of the spectra, is applied.

Finally, the spectra depicted in this work are either averages for each sample, i.e., each “simulation box”, or averages for an “atomic motif”, obtained by clustering data from similar chemical sites, as explained below. The total data set used in this study consists of approximately 2000 computational spectra.

### II.C Data Classification: Clustering of the Chemical Environments

All the atomic sites in the computational samples used in this study are grouped together (“clustered” in ML jargon) according to similarities in their local structure, in order to obtain the *fingerprint spectra*(11) of the characteristic chemical environments present in our data set. For this purpose, a many-body atomic descriptor^54^ based on the “smooth overlap of atomic positions” (SOAP)^55,56^ has been employed. SOAP descriptors encode atomic structures into a rotationally invariant numerical representation, which can then be used in ML models, e.g., to parametrize interatomic potentials^57^ or to perform data classification, as here. From these SOAP descriptors, a “kernel” function can be constructed that provides a measure of similarity between any two given atomic environments. The method used to cluster atomic environments based on these similarity scores is an unsupervised ML technique for data classification known as *k*-medoids.^58,59^ Clustering by SOAP kernels rests upon structural motifs, i.e., separating sites based on their bonding environment (bond lengths and angles) as well as on the chemical nature of the neighboring atoms. The variant of SOAP that we use, described in detail in ref (54), improves in speed and accuracy upon the standard implementation, via the introduction of modifications to the algorithm and basis functions, respectively (the basis functions in SOAP are used to numerically expand the atomic density field). Multispecies support, not described in ref (54), is added by augmenting the overall radial basis set with one basis (sub)set per species, where the bases corresponding to different species span orthogonal function spaces. All sites in the samples are clustered at the same time, with the same kernel, regardless of the central element (C, O, or H) in question. Since there are no hydrogen spectra to study, hydrogen sites in the data set are disregarded.

We find that the clustering of oxygen spectra closely recovers common “chemical intuition”. When we combine the computed X-ray spectroscopy data from all the oxygen sites in our database, we obtain fingerprints of the oxygen-containing functional groups very consistently. In the case of carbon, the situation is more complicated, since carbon is present in a wider variety of chemical environments. For this reason, SOAP hyperparameters were optimized to put more emphasis on bond lengths compared to the nature of the functional group that is possibly bonded to the carbon site. In this way, we manage to reliably separate differently bonded carbons within the sp^2^ network, i.e., distinguish between different ring structures where defects are present and estimate whether the carbon site in question is bonded with a double or single bond to the neighboring atoms.

### II.D Experimental Spectra

Experimental spectra of three samples were analyzed by utilizing computational references. Fabrication methods and the spectra themselves have been previously published in ref (30) as a part of a more comprehensive data set of carbon materials. These particular experimental samples, which are sp^2^-rich, were chosen because they are suitable to be analyzed with the computational references obtained in this work for graphene-based systems. The experimental samples were acquired as follows: highly oriented pyrolytic graphite (HOPG) was obtained from a commercial source (Scanwel, U.K.), the graphene sample was fabricated via thermal annealing, and the graphite oxide sample was made by applying a modified Hummers’ method.^30,60^

For XAS measurements, described in more detail in ref (30), a bending magnet (beamline 8–2) was used at the Stanford Synchrotron Radiation Lightsource (SSRL), employing a 55° incidence angle (magic angle) of X-ray incidence. This beamline has a spherical grating monochromator with approximately 200 meV resolution (using 40 × 40 μm^2^ slits). The total flux was on the order of 10^10^ photons/s, for which beam damage with spot size around 1 × 1 mm^2^ at the interaction point was not noticeable, even for extended exposure. The X-ray energy ranges scanned for absorption edges were as follows: carbon 1s from 260 to 350 eV and oxygen 1s from 520 to 560 eV. During all the measurements, the incoming flux was recorded using a nickel grid with a gold sputtered film. A more detailed description about the measurements as well as sample fabrication can be found in ref (30), and ref (61) provides more experimental data about these types of samples.

## III Results and Discussion

### III.A XAS Spectra of Whole Structures

The structures we employ as representative examples of oxygen-free graphene are depicted in Figure 1a–c. The defect concentration in the samples was systematically increased by creating vacancies. The C 1s XAS spectra calculated from these structures are depicted in Figures 1 and 3 for the whole samples and for selected sites around the vacancies in Figure 3b. Figure 3a shows how the features of the spectra are broadened when defect concentration increases. This is to be expected since the presence of defects breaks the symmetry of the graphene structure. In other words, the presence of defects increases the number of inequivalent atomic sites (for pristine graphene, there is only one inequivalent site). This behavior has also been shown experimentally.^5,30^

A slight shift of the π* and the σ* peaks toward higher energies can also be observed, especially in the case of the most defective sample. As discussed, the presence of the defects affects the peak positions. However, the interpretation of this broadening as observed in the calculated average spectrum of the whole sample is not straightforward. This is the reason why we also study, individually, the spectroscopic signatures of the sites around the defects themselves. Figure 3b shows how the presence of the less stable SV defect, which has a very reactive site^10^ in the middle of the larger ring structure, presents a π* feature lower in energy than that of the more stable DV defect. At the same time, the σ* feature for the SV is shifted toward higher energies than that of the DV. Compared to pristine graphene, the DV defect presents shifts of the π* and σ* features toward higher and lower energies, respectively. In contrast, the SV defect spectrum does not show appreciable shifts of these features. Instead, the main effect is a *broadening* of the peaks, accompanied by the emergence of two very small peaks at lower energies than the π*, which are related to the highly energetic dangling bond.

When the spectra from all samples are clustered, i.e., separated into different groups according to their chemical environment and, especially, their bond length, fingerprint spectra can be assigned to the different atomic motifs. Those spectra can then be flexibly combined for comparison with (and to fit) XAS and XPS data from experimental samples.

The structures presented in the study by Kumar et al.^36^ allow us to perform our XAS and XPS (Section III.C) calculations since we want to compare GO structures with varying amounts of different oxygen-containing groups systematically. Some attempts to create larger databases of ML-based GO structures have been made.^62,63^ We focus here on a more limited set of structures, because of the computational demands of full electronic-structure computations. Moreover, given the intrinsic locality of core–electron excitations, the results obtained here, within periodic boundary conditions, and utilizing data clustering, are representative of larger systems and can be directly compared with experiment. As a matter of fact, the structures presented in Figure 1 do not need to be seen as slabs, but can rather be regarded as a collection of local chemical *environments*, that will be present in experimental samples with different proportions. Furthermore, we carried out tests to verify that the results obtained from computational 2D models are also applicable to fit graphite-based experimental spectra (i.e., from 3D graphitic materials). This is because the layers in graphite do not exhibit covalent bonding, being instead bonded via weak van der Waals interactions, with correspondingly negligible chemical shifts in the X-ray spectra associated with them.

The 2D samples by Kumar et al.^36^ provide a wide variety of chemical environments, including also quite unexpected atomic environments, such as sp chains (Figure 1e,f). Similar atomic motifs have also been observed in amorphous models created with ML-based methods.^38,39^ These aforementioned models have been shown to be in very good agreement with experiment.^64,65^ Since we reclassify all the sites present in the samples *individually* to be compared with experimental spectra, also exotic chemical environments are a matter of interest since we want to understand whether those atomic motifs are present in the experimental samples. When the comparison, i.e., computational fitting, is carried out, clustered sites that are not present in the samples will not show up in the fit. Also, the convergence criteria was, naturally, kept the same for all excitation calculations and all the sites in the samples, and thus, if some sites were not physically sound enough to converge with respect to the set criteria, they were automatically removed from the data set.

Interestingly, during structural relaxation the most defective and buckled structures showed behavior resembling the so-called self-healing properties that graphene-based materials are known to have^66−69^ by closing created vacancies and forming new bonds between carbon atoms. As a result of this phenomenon these structures ended up having more disordered ring structure instead of clear SV or DV types of vacancy defects. It has been experimentally shown that vacancy defects can travel along the 2D model, they can change their nature^67^ and the resulting graphene sheet can be even completely amorphous.^67,70^ Presence of defects, not to mention lack of long-range order, changes the properties, such as conductivity and photoluminescence, of the material dramatically.^36,67−70^

The C 1s XAS spectra of oxygen-containing samples, i.e., averaged over the individual spectra of all carbon sites in the samples, are presented in Figure 4. The trends are clear: the less defective structure with less oxygen (Figure 1d) has sharp features resembling the crystalline spectra, whereas the most defective and oxidized sample (Figure 1f) has a spectrum that resembles an amorphous sample.^10,11,45^ The O 1s XAS spectra from the same samples are depicted in Figure 4. The O 1s spectra are slightly more smeared in the plots (Gaussian smearing σ = 0.5 eV) than the C 1s spectra (Gaussian smearing σ = 0.3 eV) to account for configurational disorder and thermal broadening,^11^ since the oxygen sampling is significantly smaller than carbon sampling in our structures. The same settings apply to all O 1s spectra in this work. During the fitting of the experimental spectra, in Section III.D, smearing also accounts for broadening of experimental spectra due to the finite precision of the apparatus. For fitting the spectra, a Gaussian smearing with σ = 0.5 eV is used in all cases.

As observed for the C 1s spectra in Figure 4, in the case of O 1s, there is a clear trend of how the features become broader when the amount of oxygen and the defect concentration increases and, thus, also the variety of local chemical environments increases. Figure 4 introduces an additional spectrum that was not shown in Figure 1, from a hydroxyl-rich sample (structure depicted in Figure 4c). We note that the O 1s spectra of hydroxyl-rich and ketone-rich samples differ regarding the onset of the spectra, whereas the C 1s spectra (Figure 4d) from the same samples remain nearly the same. Both samples contain hydroxyl and ketone groups, which is also likely to happen in the experimental reality. However, just by looking at the C 1s spectra it is speculative to say anything about their relative proportions. Furthermore, the features at the onset of the O 1s spectrum of the ketone-rich sample originate from the presence of ketone groups. This is evidenced by the fact that, when the oxygen sites are clustered according to their chemical environments (Figure 6), it is the ketone cluster that exhibits these features. These observations strongly suggest that, when the presence of oxygen-containing functional groups is being analyzed, most of the attention should be paid to the oxygen spectrum.

### III.B XAS Spectra of Atomic Motifs

The clustering of the sites in the computational samples was carried out by utilizing a SOAP kernel,^54−56^ as detailed in Section II.C.

**Figure 5:**
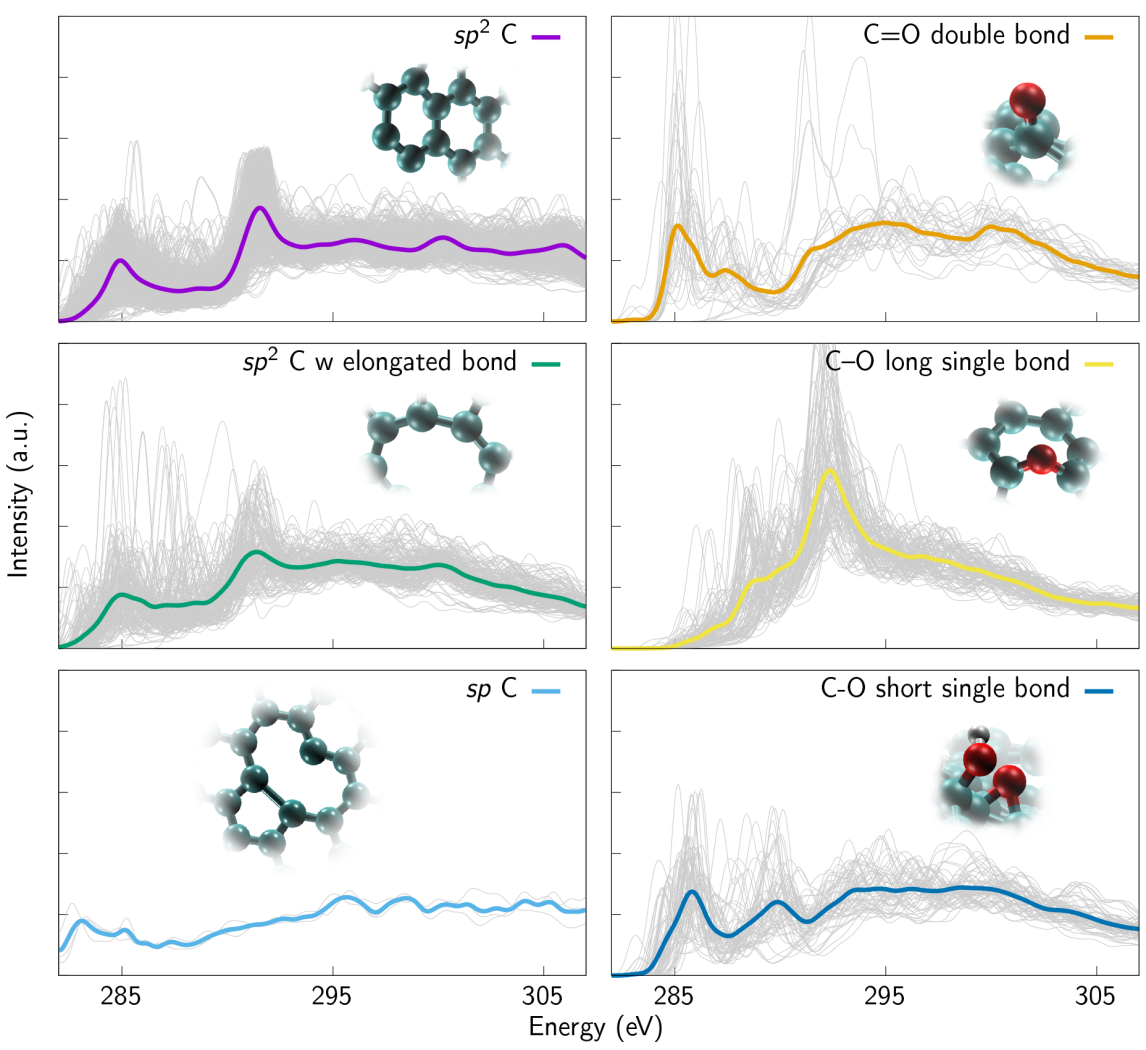
C 1s XAS spectra based on a clustering technique. All carbon sites in all samples presented in this work were clustered, i.e., classified according to their chemical environment. The individual spectra are depicted in gray, which gives an estimate of how many sites there are in each cluster and how often these sites occur in the original samples.

**Figure 6:**
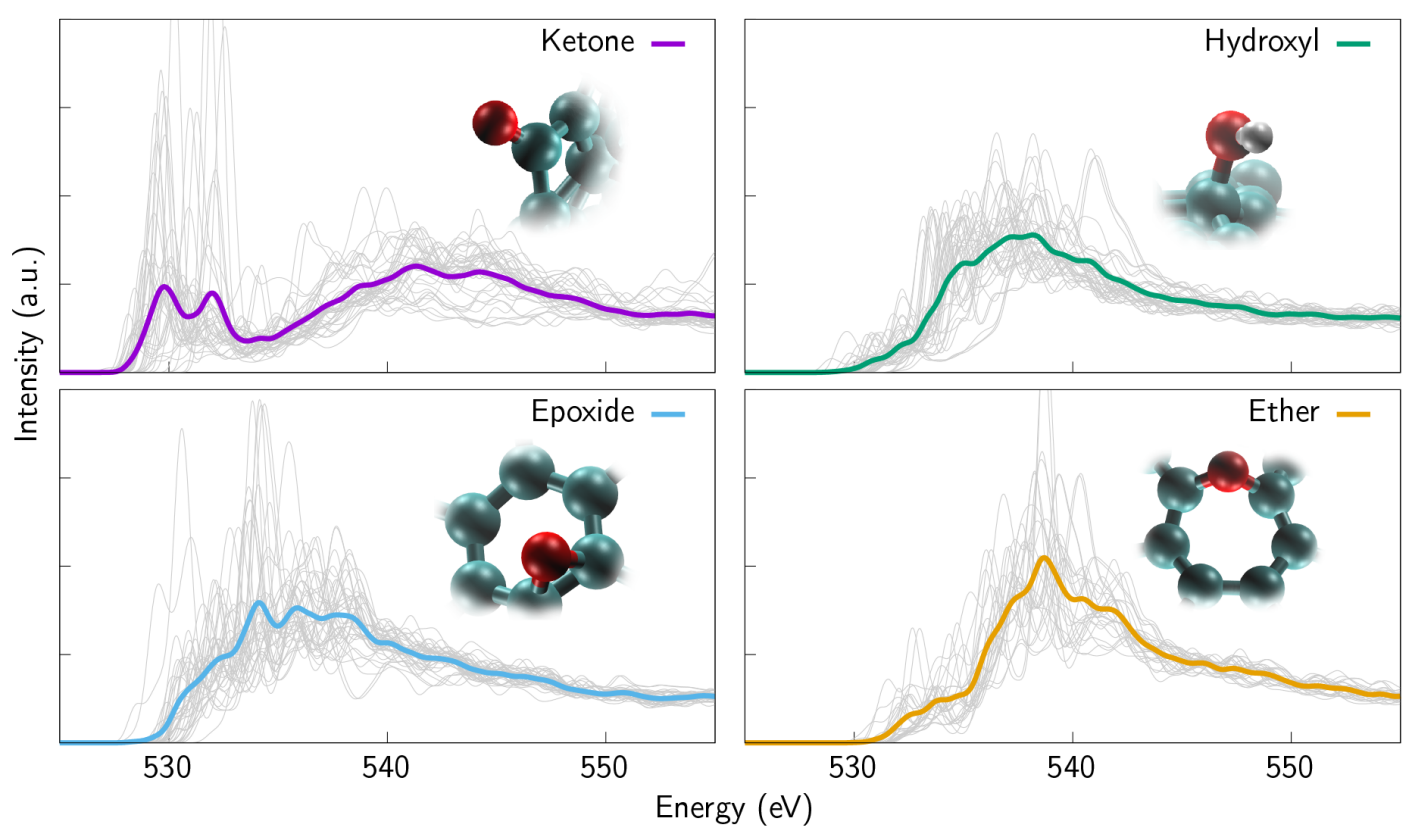
O 1s XAS spectra based on a clustering technique. Also oxygen sites in all samples presented in this work were clustered, i.e., separated from the original surroundings and classified according to their chemical environment. The individual spectra are depicted in gray, which gives an estimate of how many sites there are in each cluster and how often these sites occur in the original samples. In the case of oxygen, the clustering is in perfect agreement with the chemistry of the sites in question.

The clustered XAS spectra are presented in Figures 5 and 6. These spectra together with additional auxiliary spectra, i.e., spectra corresponding to defects or carboxylic acid (COOH) group, are used to fit the experimental spectra according to the absolute O 1s intensity measured from the sample. When the clustering was performed, emphasis was put on bond lengths over nature of neighboring atoms, although that was also taken into account. As a result, we were able to cluster oxygen sites closely following common chemical classification (ether, ketone, hydroxyl, etc.). By contrast, in the case of carbon, we can obtain more detailed information about the precise way *how* carbon atoms are bonded to their nearest neighbors. Short bond length indicates double bonding, whereas elongated bond length suggests repeated single bonding, which can be present, e.g., in bigger ring structures and in turn disrupts the sp^2^ network. In other words, this clustering scheme can distinguish between carbon bonded to oxygen with a longer single bond or with a double bond, and whether the sp^2^ network is defective.

With respect to the fitting of experimental spectra, the used approach was introduced in our previous work.^11^ However, this time we focus on carbon-based materials that contain sp^2^-bonded carbon only. As we have shown in ref (10), different types of carbon, more precisely differently bonded carbon sites, naturally have dissimilar X-ray spectroscopic signatures. Thus, in order to compare computational references with sp^2^-rich experimental samples, references obtained from sp^2^-based computational samples are necessary. Since the complexity of graphene/graphite oxide can reach the level of amorphous material, it can only be represented by a large data set of computational spectra. Then, data clustering is needed to reduce this complexity. These clustered X-ray fingerprints can be used in analysis of experimental spectra, as will be discussed in Section III.D.

### III.C Computational XPS Spectra

**Figure 7:**
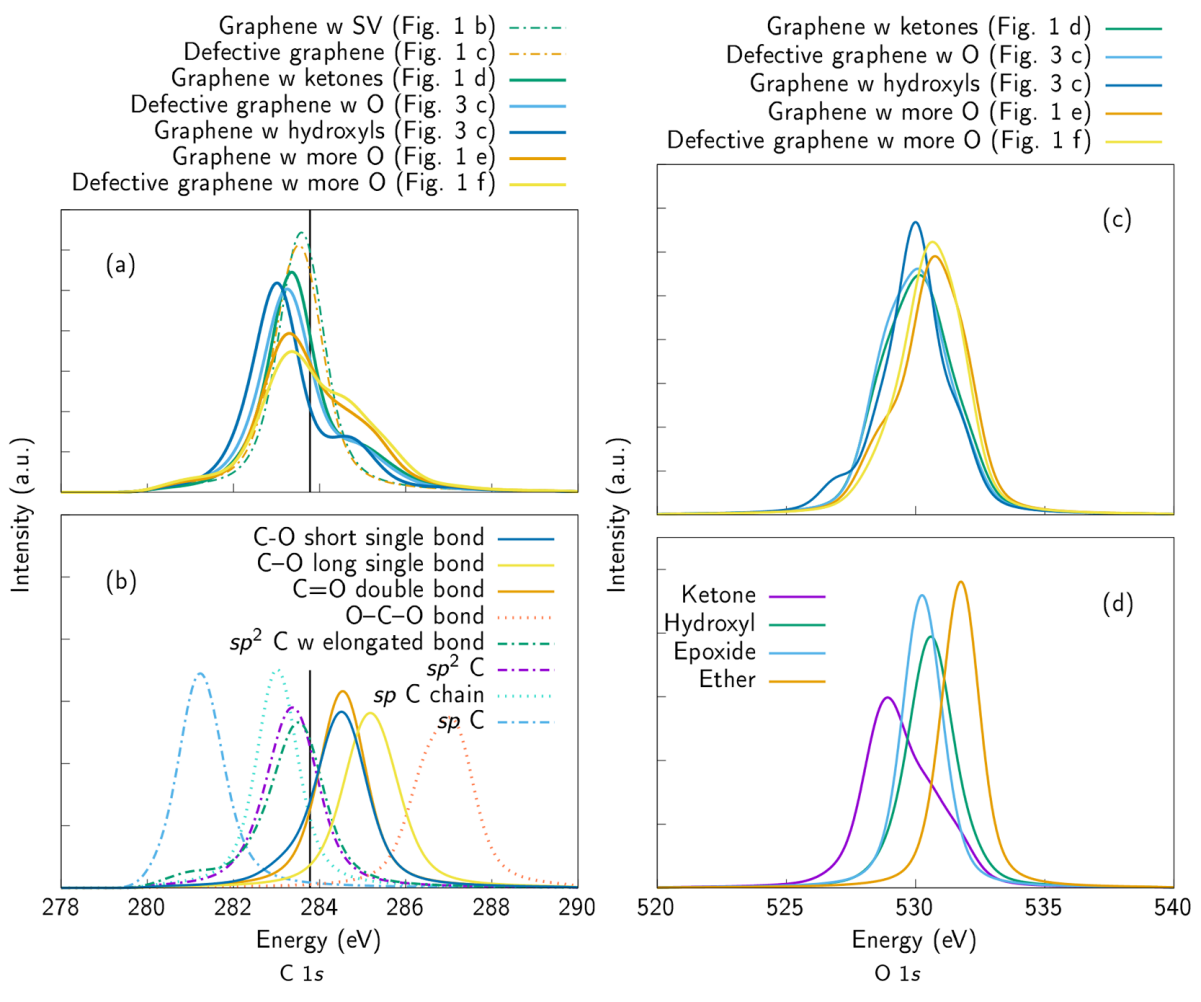
(a) Simulated C 1s XPS spectra of the graphene samples. The structures are depicted in Figures 1 and 4. The simulated XPS spectra are normalized distributions calculated from all carbon sites in the sample. (b) Clustered C 1s XPS spectra. Simulated XPS spectra are normalized distributions calculated from all clustered carbon sites. The spectra in part a can be constructed from a linear combination of these “building blocks”. The ΔKS value of pristine graphene is indicated with a vertical line. (c) Simulated O 1s XPS spectra of the oxygen-containing graphene samples (the structures are depicted in Figures 1 and 4). The simulated XPS spectra are normalized distributions calculated from all oxygen sites in the sample. (d) Clustered O 1s spectra. The spectra are normalized distributions calculated from all clustered oxygen sites and together, with different combinations, they form the spectra depicted in part c.

The calculated C 1s ΔKS distributions, that correspond to experimental XPS spectra, of the structures presented in this work are depicted in Figure 7a. The spectra for individual motifs (obtained from the clustering) are plotted in Figure 7b for comparison. The pristine graphene sample has only one C 1s ΔKS value, since all the sites are symmetry equivalent, and it is plotted with a vertical line as a reference. Note that the clusters contain two types of sp bonded carbon. The two types of sp sites in the samples arise from sites that are part of a ring (lower in energy) and sites that are part of a chain (higher in energy). The XAS calculations for chain sites did not converge, but the XPS calculations (ΔKS) did, and they are thus included in the ΔKS data set.

In this context, O–C–O refers to a carbon that is bonded to two oxygen-containing functional groups. These groups can be very different but, over all, they seem to fit in the same energy range. For instance, the computational ΔKS value for carboxylic acid (for the carbon that is bonded to the ketone and to the hydroxyl group) is approximately 286 eV, which is in good agreement with.^22^ Our results suggest that in XAS measurements this particular site shows a clear peak,^10^ whereas in XPS measurements it can be seen only as a weak tail. Experimental results support this observation.^5,6,9^

All computational XPS spectra are depicted by applying Voigtian lineshapes in order to reproduce the broadening caused by instrumental resolution (Gaussian broadening) and broadening that is caused by the lifetime of the excitation (Lorentzian broadening).^22,23^ All ΔKS distributions presented in this work are normalized.

The samples that contain only carbon (plotted with dashed lines) have narrower distributions than the samples with carbon sites that are bonded to oxygen (plotted with solid lines) (Figure 7a). Again, the simulated spectra, this time XPS spectra, show a clear trend: as the disorder increases, features are broadened. The oxygen-containing samples also have bimodal distributions, i.e., features that appear higher on the energy scale, compared to the pure carbon samples. This has also been observed experimentally.^5^ This so-called “tail” becomes higher when the amount of oxygen is increased. When we look at the clustered C 1s ΔKS distributions (Figure 7b), it is clear that these features, higher in energy, do in fact arise from carbon sites that are bonded to oxygen.

The simulated O 1s XPS spectra, depicted in Figure 7c, do not show such clear trends. Instead, the location of the main peak shifts toward higher energies as the oxygen content and the defect concentration of the sample increase. Attending to the clustered O 1s spectra (Figure 7d), this may be caused by increasing amounts of ethers within the carbon network. This would be a natural consequence of increased defect concentration, since vacancies in graphene can be reactive, and thus, enable oxygen becoming part of the ring structures.

### III.D Computational Fitting of Experimental Data

On the one hand, XAS spectra contain far more information than XPS spectra.^10,11^ On the other hand, the interpretation of both XAS and XPS experimental spectra can be equally demanding, and computational references can aid in both. However, in this work we focus on XAS fitting only, since the information captured by XPS measurement is implicitly included in XAS data in the form of energy alignment, and the measured XAS spectra are rich with features that cannot be detected with XPS.

**Figure 8:**
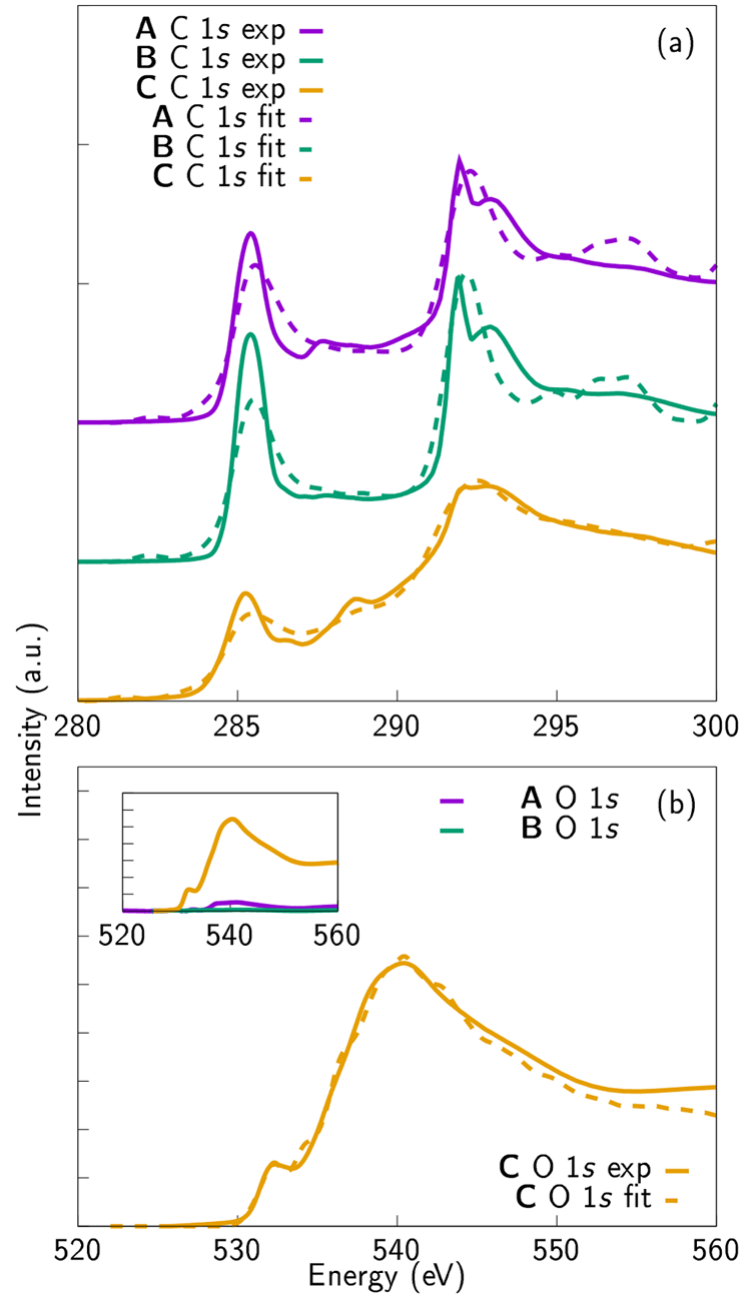
(a) Fitted experimental C 1s XAS spectra. Note that the sharp peak around 291.7 eV appears due to the excitonic effects corresponding to long-range order. (b) Fitted experimental O 1s XAS spectra. The inset in part b shows the absolute intensities of the O 1s spectra in the experimental samples. The oxygen content in samples representing graphene (**A**) and HOPG (**B**) is so low that the experimental C 1s spectra are fitted with the computational carbon database only. In the case of the GO sample (**C**), the reference spectra of carbon bonded to oxygen (C 1s) as well as oxygen reference spectra are employed (O 1s). Experimental data are taken from ref (30).

In this work we aim at using computational reference spectra to fit, and thus interpret, three experimental XAS spectra from three different sp^2^-rich samples (Figure 8). The experimental data, taken from prior work,^30^ are chosen to be representative of three different types of material: **A** is an annealed graphene sample, **B** is highly oriented pyrolytic graphite (HOPG), and **C** is a GO material. The data is fitted via the method presented in ref (11), i.e., using a linear combination of selected reference spectra. The raw data from the experimental spectra are interpolated to incorporate the same grid as the computational spectra used in the fitting. In this case, two of the experimental samples, graphene, and HOPG,^30^ contain so little oxygen that only the C 1s fitting is possible (see the inset of Figure 8b for the absolute intensities). The C 1s database, in the case of **A** and **B**, is based on clustered carbon spectra (Figure 5) from carbon sites that are not bonded to oxygen. This database is complemented with additional references from pristine graphene (Figure 3a) and from single and double vacancy sites (Figure 3b). As discussed in Section III.A, the presence of defects has a drastic effect on the properties of sp^2^-rich carbon-based materials.^36,67−70^ By using this set of selected references, we can estimate the defect concentrations in the experimental samples. The GO sample **C**,^30^ on the other hand, has substantial amounts of oxygen and, thus, also its O 1s spectrum can be fitted. For GO, we rely on a data set that is composed of all the carbon spectra presented in Figure 5, including carbon that is bonded to oxygen, as well as an auxiliary spectrum from carbon that is bonded within a carboxylic acid group. COOH seems to be a reliable reference, weakly dependent on the specific chemical surroundings.^10,11^ The COOH reference used here is computed by using a graphene-based surface with a SV and it is included separately because it is not present in the oxygen-containing computational samples, even though it is anticipated to appear in experimental samples.^5,7^

Comparison of the fitting results from the experimental samples **A** and **B** samples suggests that the defect concentration in the graphene sample is much higher than in HOPG; 70% and 40% of the bonds between the carbon atoms differ from standard sp^2^ network, respectively. The graphene sample exhibits higher amounts of SV defects (smaller ring size) as well as more elongated sp^2^ bonds (larger ring structure). This result suggests that the structure of the graphene sample is to some extent disordered and there are varying amounts of different ring sizes. The HOPG sample contains mostly pristine or nearly pristine sp^2^-bonded carbon, 60% in fact, but it is not completely free of defects either. Elongated sp^2^ bonds are not present in the HOPG fit. Both samples seem to have very small (1–2%), but not negligible, amounts of sp-bonded carbon which could appear when defects are formed or the samples are prepared for the experiments. Not being a 2D material, HOPG can be expected to be more mechanically stable than graphene and, thus, able to maintain its ordered structure while it is being handled for study. Experimental interpretation of the spectra also support these computational observations: the HOPG sample has both sharper π* and excitonic features than the graphene sample. From this computational fit, we can provide approximate estimates of the presence of certain types of defects in the samples as discussed above.

The spectrum for **C**, which is a representative of GO materials, was fitted with the oxygen-containing data set. Separate SV and DV references were excluded in this case, because having too many reference spectra makes the fitting method prone to instability. However, the sites around the defects are included in the reference data set, so we can get an estimation of the defect concentration. The fitting results suggest that the sample is highly oxidized. According to the C 1s spectrum fit, more than 60% of the carbon sites seem to be bonded to oxygen. This is in line with experimental reports based on XPS,^71,72^ which show that the amount of oxygen in some GO samples can be extremely high. We note that some of the carbons that appear, according to the fit, to be bonded to oxygen, could in fact also be bonded to other chemical species. Overlapping features caused by impurities in the experimental sample, most notably nitrogen, which are not included in the present data set, will be a topic of future research. However, experimental results show that, in the case of this particular sample, nitrogen content is very low.^30^

Nevertheless, only 10% of the carbon in the sample belongs to a regular sp^2^ network and the rest of the sites are either defective or functionalized. This can also be observed by comparing the experimental spectrum of **C** (Figure 8a) with the spectra presented in Figure 1. The closest corresponding computational model can be found in Figure 1e. In other words, by applying our fitting procedure, which is based on clustering of the structures and linear combination of the clustered spectra, we can recreate the experimental spectrum from its constituent blocks, and give more precise estimates about the composition of the sample. However, the nonoxygen related defect concentration derived from the fitting result for sample C is also high, and there is only a little pristine sp^2^-bonded carbon left. There are varying ring sizes with elongated sp^2^ bonds but also some sp-bonded carbon present.

Closer inspection of the O 1s spectrum reveals more about the distribution of oxygen-containing functional groups. Hydroxyl groups seem to dominate in this particular sample (60%), but ketones are also strongly present (20%). Ether (8%), COOH (6%), and epoxide (3%) groups appear next, in that order. Epoxides are the rarest, but still not negligible, of the groups. This is not an unreasonable conclusion, since the ring structure formed by two carbons and one oxygen is known to be unstable.^39^ These results highlight the conclusion that, when oxygen-containing functional groups are studied in more detail, most of the attention should be paid to the O 1s spectrum.

## IV Conclusions

In this work, we have computed a data set of approximately 2000 XAS spectra, and just as many ΔKS values, for simulating the XPS spectra, in order to interpret the experimental X-ray spectra of graphene and graphene/graphite oxide samples. The data will be made openly available in the near future via Zenodo. This data set can be used to understand the X-ray spectroscopy of sp^2^-rich carbon-based materials. The observed trends are as follows: the spectroscopic features are broadened as the amount of defects (either crystallographic or in the form of chemical functionalization) increases. New features appear in both XAS and XPS spectra when oxygen is present. The positions of the two main peaks that are typically exhibited by sp^2^-bonded carbon, π* and σ*, shift depending on vacancy and oxygen concentration and the nature of these defects or functionalizations. Classifying the sites in the computational samples according to their chemical environment, via ML-based clustering (or more simply, according to chemical intuition as we have done for vacancies and carboxylic acid), allows us to compare these computational spectroscopic fingerprints to experimental spectra. From these comparisons, we can make quantitative estimates of how often certain features appear in the measured spectra, and make the link with the material’s atomic structure. In addition, this method allows us to confirm whether or not simulated models are similar enough with experimental samples to be used in reliable computational experiments. Most importantly, we believe that this study will provide new insights into the characterization of sp^2^-rich carbon-based compounds, and help in the tailoring of novel materials for a variety of applications.
